# In-school versus out-of-school sedentary behavior patterns in U.S. children

**DOI:** 10.1186/s40608-016-0115-3

**Published:** 2016-07-13

**Authors:** Jimikaye Beck, Christine A. Chard, Carolin Hilzendegen, James Hill, Nanette Stroebele-Benschop

**Affiliations:** Colorado State University, 234 Gifford Building, Fort Collins, CO 80523-1571 USA; Colorado School of Public Health, Colorado State University, 1879 Sage Hall, Fort Collins, CO 80523 USA; Institute of Nutritional Medicine, University of Hohenheim, Fruwirthstr. 12, Stuttgart, 70599 Germany; Anschutz Health and Wellness Center, University of Colorado Anschutz Medical Campus, 12348 E. Montview Blvd, Aurora, CO 80247 USA

**Keywords:** Sedentary behavior, Obesity, Pediatrics, Physical activity, Accelerometers

## Abstract

**Background:**

This study contributes to the literature by using accelerometers to describe sedentary behavior (SB) patterns in US children. The purpose of this study was to examine SB patterns in fifth-graders by specifically focusing on in-school versus out-of-school SB patterns to identify when (during the school day or outside of the school day) interventions should take place in order to decrease SB in children.

**Methods:**

Data were collected from 206 fifth-graders (9–11 years old) in the Cherry Creek School District in metro Denver, Colorado (USA) during the spring of the 2010–2011 school year and fall of the 2011–2012 school year. Children wore Actical accelerometers continuously over an eight-day period. Data were analyzed using Wilcoxon rank tests, paired samples t-tests, and independent samples t-tests. Awake time was 6 AM–11 PM. We compared the percent of time spent in SB before school, during school, at recess/lunch and after school, as well as differences between boys and girls, and between children from low and high socioeconomic status schools. Children were classified as ‘non-sedentary’ or ‘sedentary’ if they participated in <360 min or ≥360 min per day of SB, respectively and were classified as ‘inactive’ or ‘active’ if they participated in <60 min or ≥ 60 min per day of MVPA, respectively.

Cross-tabs were used (and Fisher’s exact test) to identify the proportion of children in the following categories: 1) non-sedentary/inactive; 2) sedentary/inactive; 3) non-sedentary/active; and 4) sedentary/active. Statistical significance was set at *p* < 0.05.

**Results:**

All children (boys and girls and children from low and high socioeconomic status schools) participated in significantly more SB outside of school hours versus during school hours and on weekend days compared to weekdays (*p* < 0.001). Girls participated in significantly more SB than boys during weekdays (*p* = 0.015). The majority of children (65.3 %) were classified as sedentary/active.

**Conclusions:**

Given that children appear to be more sedentary during the weekend, where more opportunities to be physically active with the whole family can easily be implemented, future interventions should focus on time periods outside of school hours in order to decrease sedentary behavior and increase light physical activity in particular.

## Background

In recent years, sedentary behavior (SB) has emerged as a construct separate from that of physical activity (PA). Owen et al. and Pate et al. define SB as any waking behavior that occurs in a sitting or reclining posture and that is characterized by an energy expenditure less than 1.5 metabolic equivalents (MET) (the energy cost of physical activity, where 1 MET = 3.5 mL O_2_/kg/min) [1, 2]. Research now demonstrates that, in children, the health consequences of SB are independent from the health benefits of PA, such that achieving PA recommendations is not protective against the health risks of SB [1, 3, 4]. For example, there appears to be a bi-directional relationship between overweight/obesity and SB. Research suggests that overweight/obese children are more sedentary overall [5]. SB is also associated with greater increases in BMI over time, independent of participation in moderate-to-vigorous PA (MVPA) [6], and decreasing time spent in SB leads to reduced BMI [7].

The amount of continuous time spent in SB may be important. Saunders et al. demonstrated that, in 8–11 year old children, SB characterized by frequent interruptions was associated with a more favorable cardiometabolic risk score and lower BMI z-score as compared to SB characterized by few interruptions [8], and that children do not acutely compensate for sitting by increasing PA [9]. This lack of compensation contrasts with the concept of the ‘activitystat’, a central control mechanism hypothesized to regulate PA levels in children according to a child’s need to expend a set amount of energy throughout the day, such that children will unconsciously increase PA to compensate for SB in order to achieve sufficient energy expenditure [10–12]. One study demonstrated no relationship between the length of a sitting bout and changes in the cardiometabolic risk profile [13], somewhat supporting the ‘activitystat’ concept. Additional research is required to understand the relationship between SB and length of SB bouts with compensatory behaviors and changes in BMI and overall health risk.

Several studies in adults have also shown that, contrary to SB, light PA is associated with various health outcomes including 2-h plasma glucose [14], fasting plasma glucose [15], waist circumference [15, 16], BMI [15], insulin sensitivity [16], and other cardiometabolic risk factors [15]. Additionally, two studies, one in adults [17] and the other in adolescents [18], have shown that reallocating SB to light PA results in positive changes in cardiometabolic risk factors, including decreased triglycerides and insulin [17] and lower diastolic blood pressure and HDL-cholesterol [18]. While more research is needed to determine the role of light PA in cardiometabolic health, studies to date suggest that shifting from SB to light physical activity may be sufficient for promoting positive cardiometabolic health benefits.

While the associations between both SB and light PA and cardiometabolic disease risk have been studied to some extent [8, 9, 13–18], limited data are available characterizing SB patterns in children throughout the entire day. Current data indicate that 6–11 year old children in the United States (US) engage in SB for between 6 and 8 h per day [19, 20] with children from Canada and the United Kingdom (UK) spending similar amounts of time in SB (6–7.7 h/day) [5, 21, 22]. Many of these studies relied on either self-reported time spent in sedentary activities (such as TV viewing, video-game playing, computer use, etc.) [7] or on the use of accelerometer data measured in one-minute epochs [5, 6, 19–21, 23]. The use of one-minute epochs can lead to classification errors by washing-out the ability to identify short-bursts of moderate or vigorous activity during the one-minute period [24]. Therefore, shorter epoch lengths are more appropriate for examining and classifying SB patterns in children [24]. We identified only two studies, both from the UK, using accelerometers with 5-s epochs to describe children’s SB and PA patterns throughout the week versus the weekend [22, 25]. Results from those studies were mixed with Steele et al.’s study suggesting that children spent more time in SB on weekends versus weekdays [22]. Fairclough et al.’s study found that highly active boys and girls exhibited no differences in SB on weekdays versus weekends; however, similar to Steele et al.’s findings, children in lower quartiles of physical activity spent more time in SB on weekends compared to weekdays [25]. Additional research is required to understand children’s SB patterns in the US, particularly SB patterns during specific time periods throughout the week (such as the school day, PE time, recess, etc.) and weekend time.

Our study contributes to the literature by using accelerometers to describe SB patterns in US children throughout the week and weekend, including various time periods during and outside of school hours. The purpose of this study was to examine SB patterns in fifth-graders (9–11 years old) to better inform the development of interventions aiming to decrease child SB, with the ultimate goal of improving child health by attenuating the negative effects of SB on BMI and cardiometabolic health [3, 5, 6, 8, 9, 13]. This study specifically focuses on in-school versus out-of-school SB patterns to identify when (during the school day or outside of the school day) interventions should take place in order to decrease SB in children.

## Methods

### Participants

Data were collected from 206 fifth-graders (9–11 years old) in the Cherry Creek School District (CCSD) in metro Denver, Colorado (USA) during the spring of the 2010–2011 school year and fall of the 2011–2012 school year. A convenience sample of eleven elementary schools were selected from a total of 42 elementary schools based on the rates of free or reduced lunch eligibility within the school. Children qualify for FRL if their household income is 1.30 times (reduced lunch) or 1.85 times (free lunch) the federal poverty guidelines. CCSD personnel provided school-level FRL eligibility rates to researchers. One fifth-grade classroom from each school participated in the study. There are an average of 24 children per fifth-grade classroom (approximately 77 % of eligible children participated in the study).

### Instruments

Habitual free-living SB was measured using accelerometers (Actical B series, software version 3.0, Philips Electronics, Oregon).

### Procedure

One fifth-grade class from each elementary school was invited to participate in accelerometer data collection. The children were instructed to wear the accelerometer continuously over an eight-day period. The device measures 29 mm x 37 mm x 11 mm and weighs 22 g with a battery and wrist band. The accelerometer was attached to the wrist of the child’s non-dominant hand with a medical band. The Actical B-series Physical Activity Monitor is a non-invasive, waterproof, omni-directional accelerometer that records acceleration data in 15-s epochs. Fifth-grade teachers were asked to complete a class schedule for the dates during which their fifth-graders wore the accelerometers. The class schedule included the following information: 1) school start time; 2) school end time; 3) recess/lunch start time; 4) recess/lunch end time; 5) the day(s) of the week their fifth graders had physical education (PE) class; 6) time of day PE class started; and 7) time of day PE class ended. Recess and lunch are a combined time period where children come and go from lunch as they are ready, depending on whether lunch is before or after recess; therefore, we were unable to separate lunch time from recess time in the analyses.

Demographic information was collected through multiple sources. The percent of children qualifying for free or reduced lunch (FRL) at each school was used as a proxy measure for socioeconomic status (SES). Schools were considered to have ‘low’ SES if they attended a school where 40 % or more of students qualified for FRL, and were considered to have ‘high’ SES if they attended a school where less than 40 % of students qualified for FRL. Individual child gender and age were determined before attaching the accelerometer to the child’s wrist by asking children to complete a short survey indicating their gender and age.

### Data analysis

Awake time for accelerometer data was classified as 6 AM-11 PM. Data were considered missing if there were more than 20 min of consecutive zeros [25]. If more than 10 % of data within a defined time frame (e.g., before school or recess/lunch) were missing, data from the time frame were not included in the analyses. Three out of five weekdays or at least sixty-percent of data for all time frames of all weekdays were required to be included in the analyses of weekdays. For comparing data during school time versus outside of school time at least 3 of 5 days of data were required [25]. Additionally, for comparing data during school time, both recess and school day data had to be available and, for comparing data outside of school time, both before and after school data had to be available in order to be included in the analyses. If there were more than 120 min of data missing on a weekend day, that day was not included in the analyses. In addition to these criteria, accelerometer data for the weekend were only analyzed for fifth-graders who provided data for at least one complete weekend day. Data from accelerometers that malfunctioned (*n* = 3) or for which only sporadic data were available (*n* = 7) were not included in the analyses. Based on each of these criteria, data from 196 of the original 206 fifth graders were analyzed.

Accelerometer data were broken down into distinct time periods based on class schedules provided by fifth-grade teachers. These time periods included before school, the entire school day, recess/lunch, PE class, and after school. The time period for PE class was removed from final data analyses because, following initial analyses, the times and dates provided by fifth-grade teachers regarding PE class were inconsistent with accelerometer counts (e.g., children were completely sedentary during the identified PE dates/times), and researchers were unable to confirm the actual times and dates during which PE occurred. Percent of time fifth-graders spent in sedentary, light, moderate and vigorous PA were determined for each of the distinct time periods, as well as for entire weekdays and weekend days. Percent of time was used instead of minutes in order to compare between groups due to the fact that the distinct time periods were different lengths of time depending on the school the children attended (e.g., recess/lunch duration ranged from 20 to 45 min). Cut-points for sedentary, light, moderate and vigorous PA were determined based on research by Heil [26]. Data were analyzed for all fifth-graders, as well as run separately for sex and SES. Children were classified as ‘non-sedentary’ or ‘sedentary’ if they participated in <360 min or ≥360 min per day of SB, respectively (based on findings that U.S. children spend an average of 6 h per day in SB [19]) and were classified as ‘inactive’ or ‘active’ if they participated in <60 min or ≥ 60 min per day of MVPA, respectively.

The data were analyzed using SPSS version 22. Friedman tests were used to compare activity levels during the four distinct time periods analyzed (before school, school day, recess/lunch, after school). Paired samples t-tests or Wilcoxon rank tests were used to compare amount of time spent in SB and light PA during school versus out-of-school hours and during weekdays versus weekend days for all children, within boys and girls and within children from low and high SES schools. Independent samples t-tests were used to compare the amount of time spent in SB and light PA during the weekdays and weekend days between boys and girls and between children from low versus high SES schools. Cross-tabs were used (and Fisher’s exact test) to identify the proportion of children in the following categories: 1) non-sedentary/inactive; 2) sedentary/inactive; 3) non-sedentary/active; and 4) sedentary/active. Statistical significance was set at *p* < 0.05.

## Results

Of the 196 children studied, 50.5 % were male, 48.5 % were female and 1.0 % did not report sex. The average age of the children was 10.5 ± 0.53 years. Additionally, 24.5 % of the children (*N* = 48) attended low SES schools, while the remaining 75.5 % of children (*N* = 148) attended high SES schools.

**Fig. 1:**
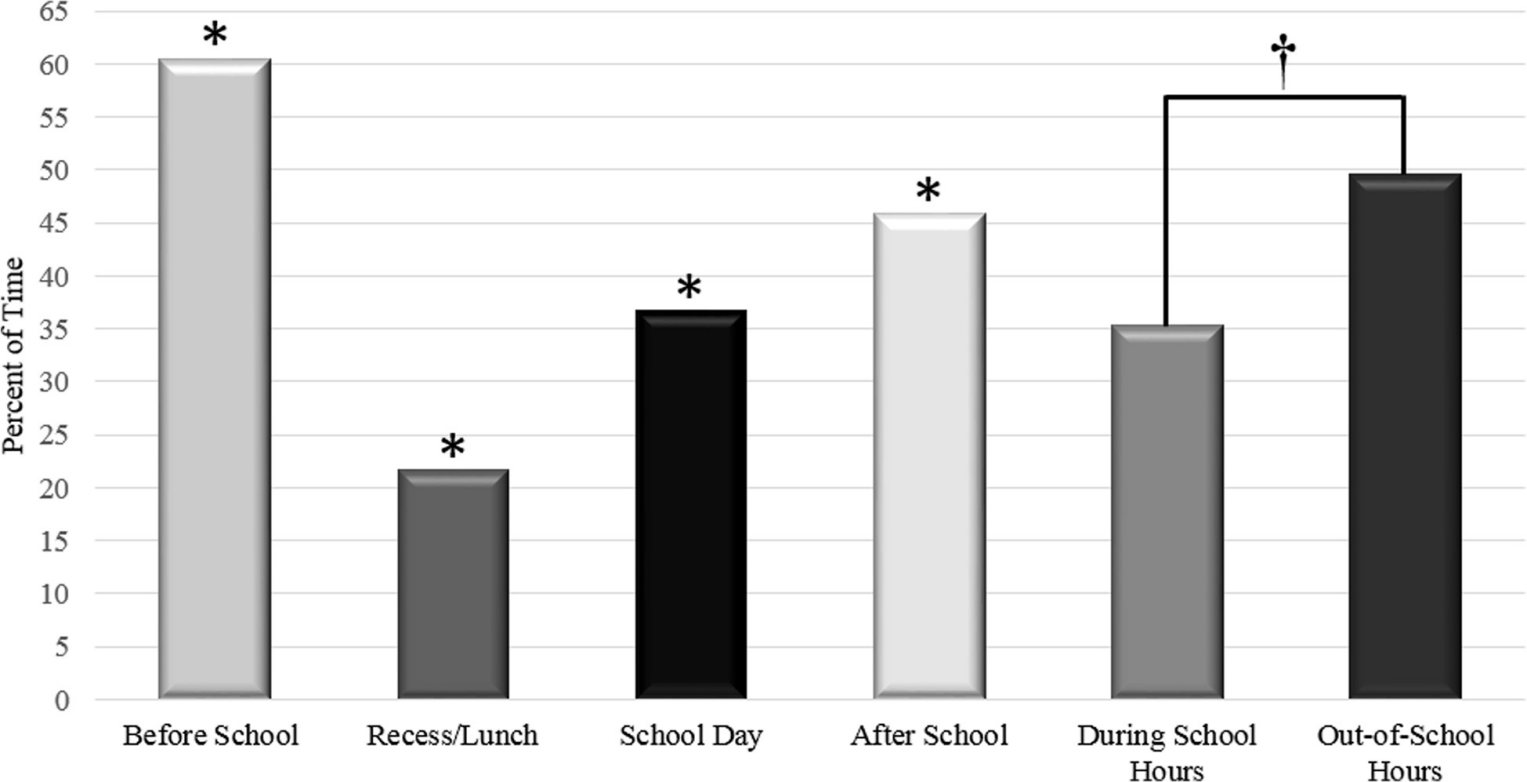
*p < 0.001 for differences over all four time frames: before school, recess/lunch, school day, and after school. †*p* < 0.001 for difference between during school hours and out-of-school hours

Figure 1 shows the percent of time children spent in SB throughout the day, broken down into four distinct time periods (before school, school day, recess/lunch and after school), as well as during school hours versus out-of-school hours.

Children spent a significantly greater proportion of their time in SB before and after school when compared to both the school day and recess/lunch time (Friedman test: *p* < 0.001). Children spent the smallest proportion of their time (21.7 %) in SB during the recess/lunch time period. Additionally, children spent a significantly smaller proportion of their time in SB during school hours versus out-of-school hours (Paired *t*-test: *p* < 0.001).

**Fig. 2:**
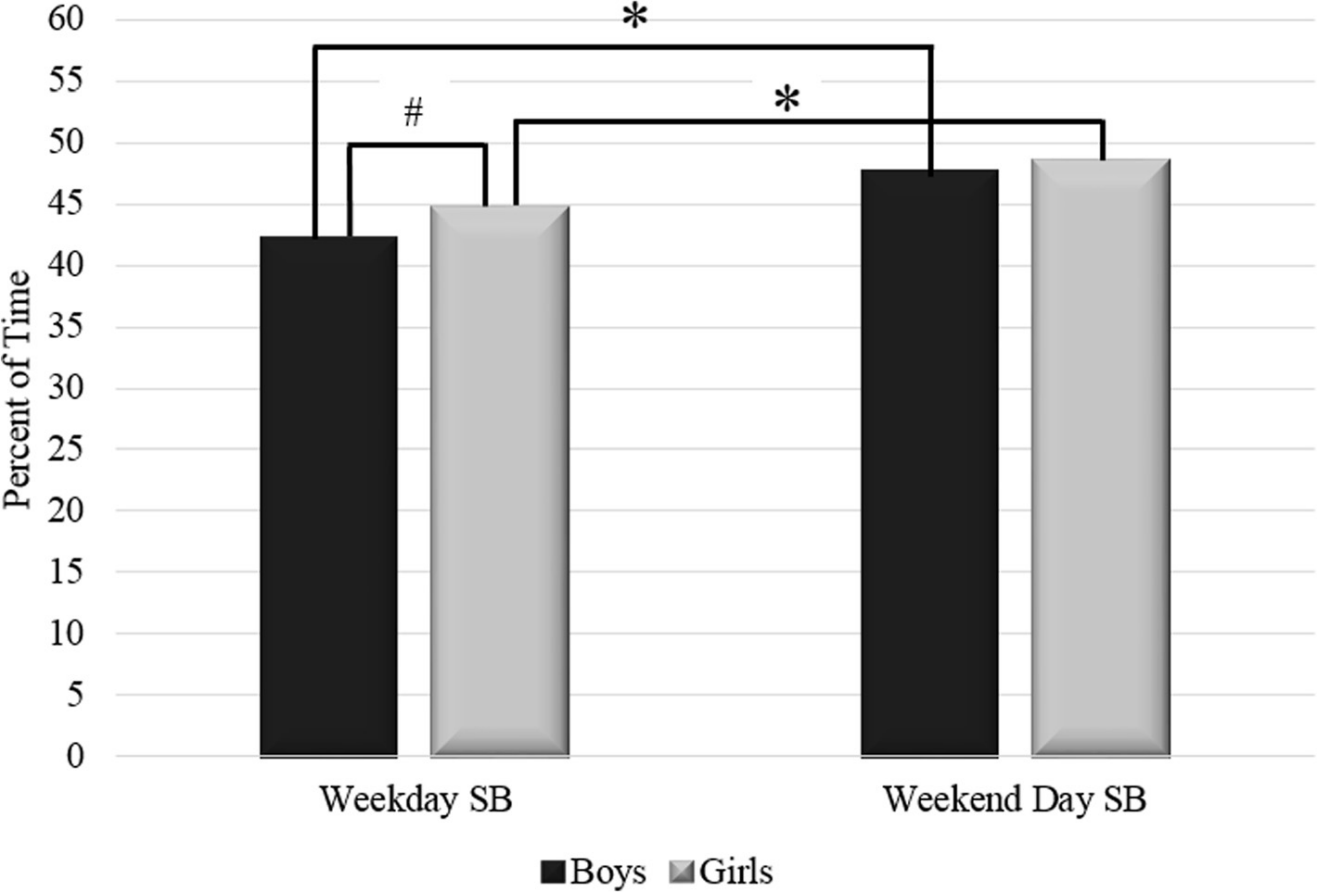
*p < 0.001 for within gender differences between weekdays and weekend days. #*p* = 0.015 for difference between boys and girls during weekdays

Figure 2 compares the average percent of time spent in SB during weekdays versus weekend days stratified by sex.

Both boys and girls spent a significantly greater proportion of their time in SB on weekend days versus weekdays (Paired *t*-test: *p* < 0.001), with girls spending a significantly greater proportion of their weekday time in SB compared to boys (Independent *t*-test: *p* = 0.015). Girls and boys spent similar proportions of their weekend time in SB (Independent *t*-test: *p* = 0.397).

**Fig. 3:**
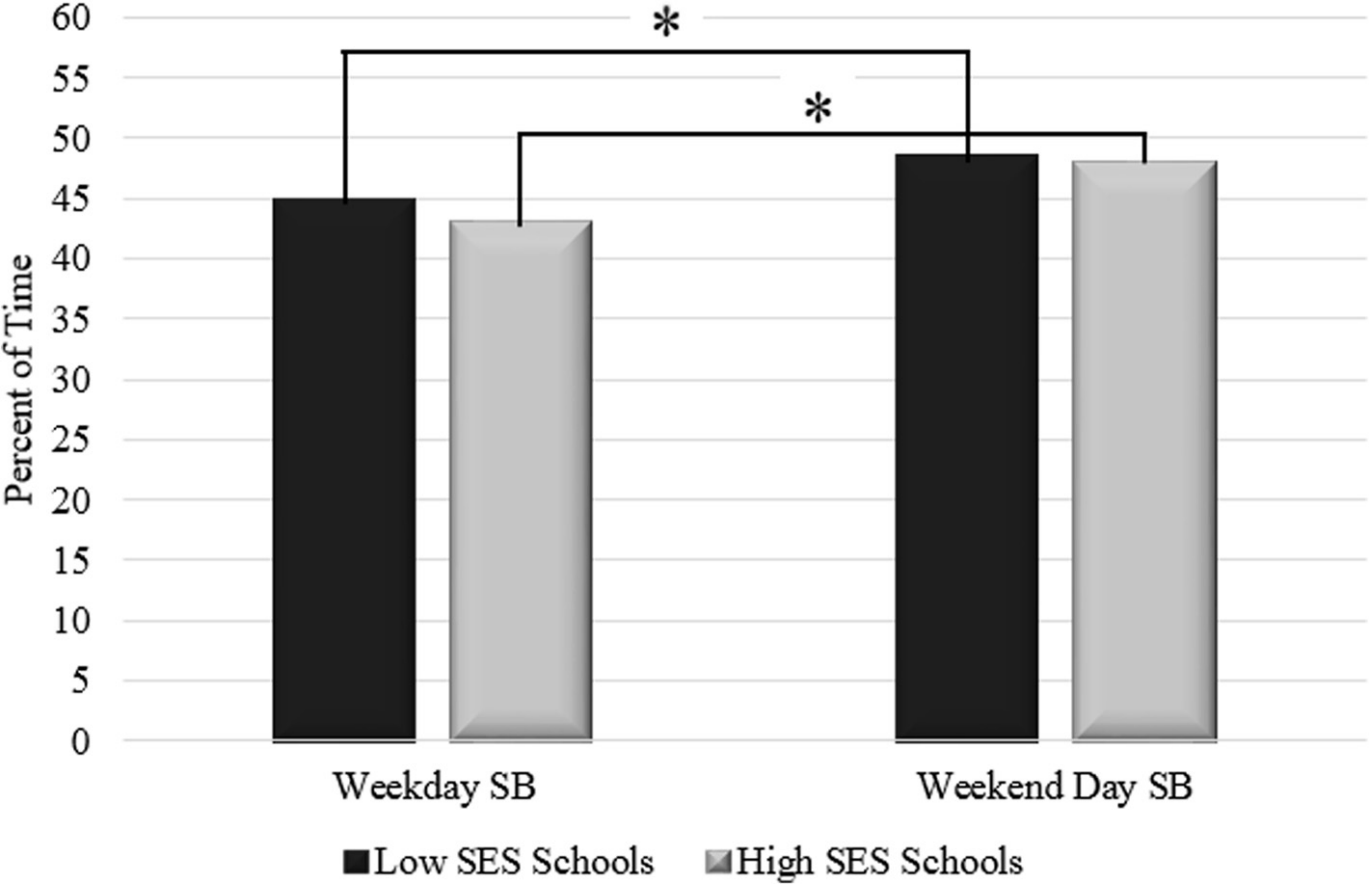
*p < 0.001 for within SES differences between weekdays and weekend days

Figure 3 shows the average percent of time spent in SB on weekdays versus weekend days stratified by children in low (≥40 % FRL) versus high (<40 % FRL) SES schools.

Children from both low and high SES schools spent similar proportions of their weekdays and weekend days in SB. Both groups demonstrated significant increases in the proportion of time spent in SB during weekend days compared to weekdays (Paired *t*-test: low SES: *p* = 0.001; high SES: *p* < 0.001).

**Fig. 4:**
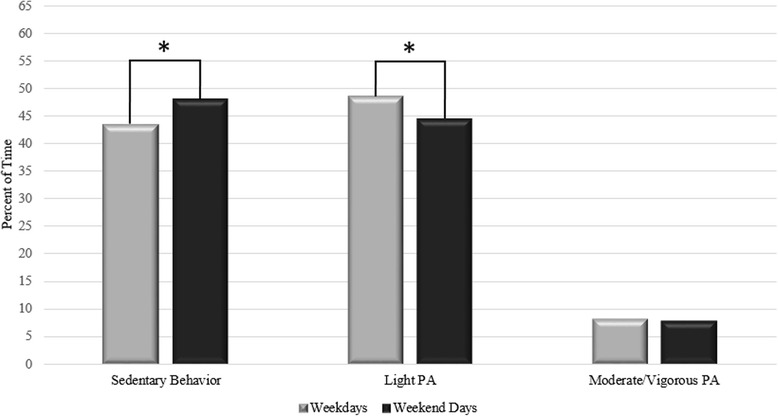
*p < 0.001 for differences between weekdays and weekend days

Figure 4 shows the average percent of time spent in SB, light PA, and MVPA on weekdays versus weekend days.

As shown, children spent significantly less time in SB on weekdays versus weekend days (43.4 and 48.0 %, respectively; Paired *t*-test: *p* < 0.001) and spent significantly more time in light PA on weekdays versus weekend days (48.6 and 44.3 %, respectively; Paired *t*-test: *p* < 0.001). Time spent in MVPA did not vary significantly on weekdays versus weekend days (8.0 and 7.7 %, respectively; Wilcoxon ranks test: *p* = 0.072).

**Fig. 5:**
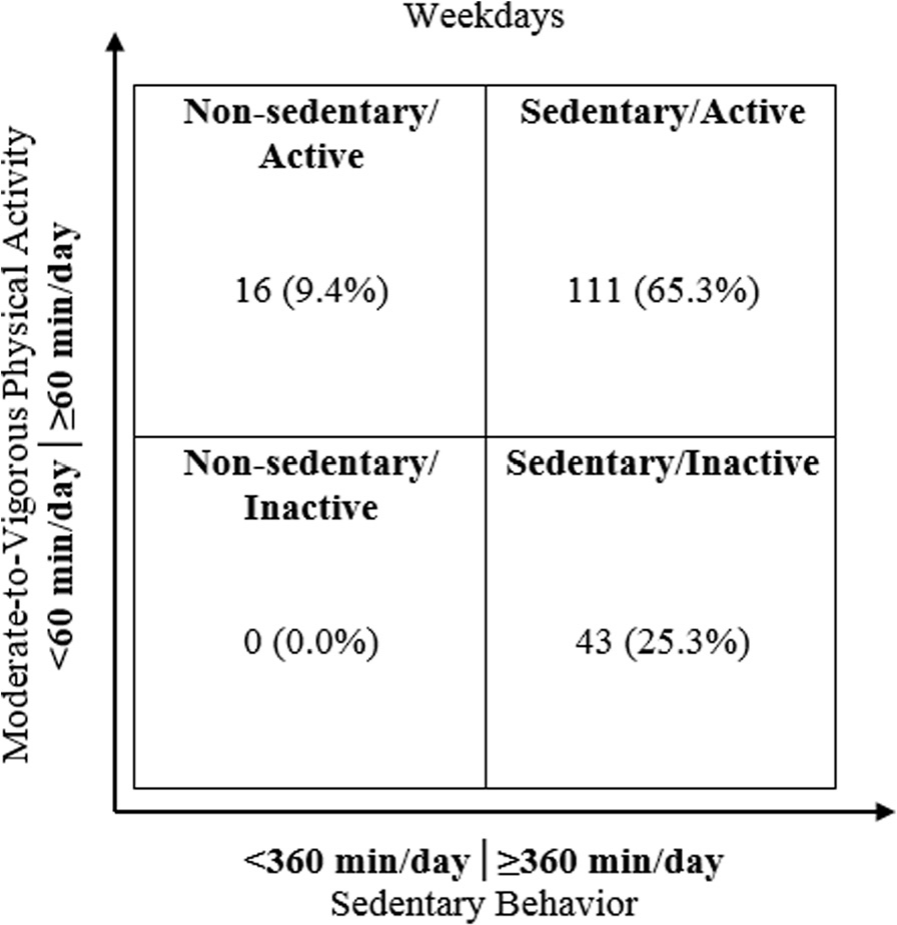
Amount of weekday time spent in ‘Low’ or ‘High’ amounts of Sedentary Behavior (SB) and Moderate-to-Vigorous Physical Activity (MVPA)

Figure 5 shows the cross-tabs results from the weekday analyses, with children categorized based on the amount of time spent both in SB and in MVPA.

As shown, the majority of children (*N* = 111, 65.3 %) were categorized as sedentary/active. One-quarter of children (*N* = 43, 25.3 %) were in the sedentary/inactive category, with fewer children in the non-sedentary/active group (*N* = 16, 9.4 %) and no children in the non-sedentary/inactive group (*N* = 0, 0.0 %). Cross-tabs analyses revealed that children who participated in large amounts of SB were significantly more likely to also exhibit small amounts of MVPA (*p* = 0.013).

## Discussion

This study used accelerometers to examine SB patterns of US children during different time periods throughout the school day and outside of school hours. Our findings revealed that the children in this study participated in significantly more SB outside of school hours versus during school hours, as well as on weekend days compared to weekdays. Girls participated in significantly more SB on weekdays than did boys. Importantly, all of the children in this study, both boys and girls, as well as children from both low and high SES schools, demonstrated significant increases in SB on weekend days and outside of school hours compared to weekdays and during school hours. Finally, the majority of children were classified as participating both in high amounts of SB and high amounts of MVPA (sedentary/active).

Our finding that girls spent a greater proportion of their weekday time in SB than boys is consistent with recent studies suggesting that boys spent slightly less time in SB than girls [19, 20, 22, 27]. Additionally, the finding that our children participated in significantly more SB outside of school hours was consistent with findings from Steele et al. [22]. However, Fairclough et al. found that differences in SB from weekdays to weekends were dependent on the child’s sex and habitual level of activity [28]. Specifically, Fairclough et al. found that SB increased on the weekends in all boys except those in the highest PA quartile, who demonstrated minimal variation in SB across weekdays versus weekends [28]. In contrast to our findings, girls in the middle two quartiles of PA participated in greater amounts of SB on weekdays compared to weekends, with girls in the highest PA quartile participating in similar amounts of SB on both weekdays and weekends [28]. We used quartiles to examine our data (data not shown) in order to compare our findings to those of Fairclough et al. and found that differences still existed when using quartiles of PA. It is possible that the differences are due to the populations studied, as our children were from the U.S. and Fairclough et al.’s children were from England. Nonetheless, additional research is required to better understand children’s SB patterns on weekdays versus weekends, with a particular need for examining sex differences in SB patterns during these time periods.

In addition to examining differences between weekdays and weekend days, the use of accelerometers allowed us to classify the proportion of time children spent in SB during distinct time periods throughout the weekdays. Our results showed that children achieved their lowest percent of time spent in SB (21.7 %) during the recess/lunch time period. While we were unable to identify other studies examining SB patterns during specific time periods throughout the weekdays, this finding was not surprising, as other studies have demonstrated that children spend a large percentage of their recess time in MVPA [29, 30]. As such, one would expect correspondingly lower levels of SB. Our data also revealed that children only spent 36.7 % of their time at school in SB, while they spent a significantly greater proportion of their before and after school time in SB (60.4 and 45.8 %; *p* < 0.001). These findings imply that interventions aiming to decrease SB should consider focusing on before and after school time periods, as well as weekends, since these time periods appear to have the greatest room for changing SB.

In conjunction with our findings that SB increases outside of school hours, we also found that increasing SB corresponded with decreasing light PA. Specifically, children in our study spent a significantly greater proportion of their time in light PA on weekdays compared to weekend days (48.6 and 44.3 %, respectively), while time spent in MVPA varied little on weekdays versus weekend days (8.0 and 7.7 %, respectively). These findings were true regardless of sex or SES, with the exception of low SES children, who spent a significantly greater proportion of their time in MVPA on weekdays compared to weekend days (8.0 % vs. 6.3 %, respectively, Wilcoxon ranks test: *p* = 0.005). When examined as a whole these findings indicate that children shifted away from light PA during weekdays towards greater SB on the weekends. This shift from light PA to SB is concerning due to growing evidence that the health consequences of SB, including a less healthy cardiometabolic profile, are independent from participation in MVPA [1, 3, 4, 13], and due to the fact that light PA is independently associated with cardiometabolic health benefits [14–18].

The shift from light PA to SB is also concerning considering recent research indicating that children do not compensate for SB by increasing PA levels [9], which contrasts with the previously described ‘activitystat’ idea [10–12]. Indeed, our findings also contrast with the ‘activitystat’ idea, as our children did not compensate for increased SB on the weekends by increasing either light PA or MVPA. These contrasting findings could be due to methodological differences regarding how Wilkin et al. (2006) and Fremeaux et al. (2011) categorized their accelerometer data. Wilkin et al. categorized their data as ‘low’, ‘medium’ and ‘high’ intensity [10], while Fremeaux et al. categorized their data as total physical activity or MVPA [11]. In both cases the researchers neglected to identify SB separately from light PA. Combining SB with light PA may result in inaccurately representing SB as light PA, thus preventing a clear understanding of children’s activity behaviors at the low end of the activity spectrum (e.g., SB and light PA). This also ignores the differing health consequences of SB versus light PA. The lack of a distinct SB category prevents a direct comparison with our data. Regardless, our finding that children shift away from light PA and towards SB (while maintaining MVPA levels) suggests that perhaps, instead of targeting MVPA, interventions should potentially focus on decreasing SB by shifting towards light PA, as suggested by other researchers as well [6, 14–18, 20, 31].

Additional data from our study support the notion that, instead of targeting MVPA, future interventions should consider focusing on replacing SB with light PA. We categorized children based on the amount of time spent in both SB and MVPA. Children were classified as either ‘sedentary’ (≥360 min/day in SB) or ‘non-sedentary’ (<360 min/day in SB) and as either ‘active’ (≥60 min/day in MVPA) or ‘inactive’ (<60 min/day in MVPA). We found that the majority of children (65.3 %) were classified as sedentary/active, 25.3 % of children were classified as sedentary/inactive and the remaining 9.4 % of children classified as non-sedentary/active. These findings indicate that, while a large proportion of children were achieving recommended amounts of MVPA [32], they also spent a significant amount of their day in SB. Our findings contrast somewhat with those of Herman et al. [5]. In their study, a smaller percentage of boys and girls were classified as active/sedentary (27.6 and 5.2 %, respectively), with a slightly larger percentage of boys and girls classified as inactive/sedentary (34.3 and 38.7 %, respectively) and active/non-sedentary (18.5 and 10.1 %, respectively) [5]. These differences could be due to the classification criteria, as Herman et al. used screen time to classify sedentary behavior (>2 h/day = ‘sedentary’, ≤2 h/day = ‘non-sedentary’), while the current study used total minutes spent in SB. Despite these differences, it is concerning that the majority of children in both studies were classified as sedentary. Due to the fact that the health and obesity risks associated with SB exist independently from MVPA [1, 3, 4, 6, 8], greater emphasis should be placed on decreasing children’s SB, as opposed to simply increasing MVPA. The fact that PA interventions in children have been largely unsuccessful in achieving long-term changes in BMI [33] and PA [34] also suggests that future interventions should perhaps focus on a potentially more achievable target- transitioning children from SB to light PA, as has been suggested by other researchers [6, 14–18, 20, 31].

### Limitations

There were some limitations to this study. We classified awake time as 6 AM to 11 PM. This may have resulted in misclassifying sleep time as SB, which would result in overestimating SB. Future studies should include records of sleep time to prevent misclassification of sleep time as SB. We used 360 min of SB per day to classify children as either sedentary or non-sedentary based on a review determining that children aged 6–11 years spend an average of six hours per day in SB (Pate 2011), and due to the fact, at the time of data analyses, no other studies we found had dichotomized children based on SB. Additional research is required to determine the best cut-point for identifying children as sedentary or non-sedentary. We did not classify the type of SB in which children participated, which prevented a more specific representation of children’s overall activity behaviors. Some researchers have advocated for the concurrent use of objective measures of SB (accelerometers) with self- or proxy-report tools in order to identify both the type and amount of SB [35–37]. The original purpose of this study did not include specifically classifying the type of SB; however, future studies should include the type of SB in order to better understand the specifics of children’s sedentary behaviors, thereby allowing more targeted interventions [7, 9]. The students in our sample attended a fairly affluent school district in the Denver-metro area; therefore, the generalizability of our findings is limited to this and other similar districts throughout Colorado and the US. Finally, this study should be replicated in a larger sample of children and in a variety of socioeconomic settings. In spite of these limitations, we believe that our findings contribute to the literature because we are one of very few studies that have used accelerometers with short epoch lengths (15-s) to examine SB patterns in US children during specific time periods throughout the school day.

## Conclusions

Given that children appear to be more sedentary outside of school hours, particularly during the weekend (a time period during which more opportunities to be physically active with the whole family can be easily implemented), future interventions should focus on time periods outside of school hours in order to decrease sedentary behavior and increase light, moderate, and vigorous physical activity. Our findings revealed that children specifically shifted from light PA to SB outside of school hours and that these shifts occurred in both boys and girls, as well as among children from both low and high SES schools. We suggest that future interventions should consider focusing on shifting from SB to light PA [7, 17, 18, 20, 31], which may be sufficient for overcoming the health and obesity risks associated with SB [14–18] and could increase the likelihood for a successful intervention. Such interventions could help reduce long-term health and obesity risk through a focus on decreasing SB and increasing light PA in children.

## Abbreviations

CCSD, Cherry Creek School District; FRL, free or reduced lunch; MET, metabolic equivalents; MVPA, moderate-to-vigorous physical activity; PA, physical activity; PE, physical education; SB, sedentary behavior; SES, socioeconomic status; UK, United Kingdom; US, United States
